# Animal Models of Cystic Fibrosis Pathology: Phenotypic Parallels and Divergences

**DOI:** 10.1155/2016/5258727

**Published:** 2016-06-01

**Authors:** Gillian M. Lavelle, Michelle M. White, Niall Browne, Noel G. McElvaney, Emer P. Reeves

**Affiliations:** Respiratory Research Division, Department of Medicine, Royal College of Surgeons in Ireland, Beaumont Hospital, Dublin 9, Ireland

## Abstract

Cystic fibrosis (CF) is caused by mutations in the cystic fibrosis transmembrane conductance regulator (CFTR) gene. The resultant characteristic ion transport defect results in decreased mucociliary clearance, bacterial colonisation, and chronic neutrophil-dominated inflammation. Much knowledge surrounding the pathophysiology of the disease has been gained through the generation of animal models, despite inherent limitations in each. The failure of certain mouse models to recapitulate the phenotypic manifestations of human disease has initiated the generation of larger animals in which to study CF, including the pig and the ferret. This review will summarise the basic phenotypes of three animal models and describe the contributions of such animal studies to our current understanding of CF.

## 1. Introduction

*The Multiorgan Pathology of Cystic Fibrosis.* In 1989 the CFTR gene was identified and isolated from epithelial cells of the pancreas, lungs, colon, sweat glands, and nasal polyps of healthy control individuals [1, 2]. By facilitating ion transport into and out of the cell, CFTR function results in hydration of the airways and normal airway surface liquid (ASL) volume. Adequate ASL permits optimal movement of airway cilia, whose function is to remove mucus containing proinflammatory mediators, immune cells, and inhaled pathogens from the lung. CF is an autosomal recessive inherited disorder caused by mutations within the CFTR gene [2], leading to increased morbidity at a young age [3]. CF affects 70,000 people worldwide [4] and is characterised by defective CFTR function, resulting in decreased chloride (Cl^−^) secretion and hyperabsorption of sodium (Na^+^). Reduced ion transport significantly dehydrates the airway mucus leading to reduced ASL, preventing adequate removal of mucus* via* the mucociliary escalator [5]. Mucus-laden cilia become dyskinetic with the resultant pathological triad that hallmarks CF: chronic airway mucus build-up, microbe trapping, and sustained inflammation involving persistent inflammatory cell influx to the lungs, leading to pulmonary function loss and poorer clinical outcome [6, 7].

Although the chronic pathology of the lung represents the most serious clinical manifestation [8, 9], CF is a multiorgan disease. Extrapulmonary clinical manifestations of CF include impairment of the gastrointestinal (GI) tract. For example, meconium ileus (MI), a condition caused by increased viscosity of the intestinal mucus within hours of birth, occurs in 13% to 17% of infants with CF [10]. The CFTR protein is also expressed on the apical membrane of pancreatic epithelial cells. Here, it modulates Cl^−^ absorption and secretion of bicarbonate, an important buffer for maintaining optimum pH. Inspissated mucus obstruction, secondary to dysfunctional CFTR in the pancreas, perpetuates localised inflammation and pancreatic scarring. Common pathologies associated with this organ in CF include pancreatic insufficiency, pancreatitis, glucose intolerance, and CF-related diabetes mellitus [11]. In this regard, CFTR plays an important role in the secretion of pancreatic proenzymes (e.g., zymogen) into the duodenum and poor endocrine function, as occurs in CF, results in malabsorption of nutrients including fat-soluble vitamins, cholesterol, and proteins, leading to poor weight gain and nutritional deficiency. CF can also affect the hepatobiliary system. Liver disease is caused by biliary obstruction and progressive periportal fibrosis, resulting in biliary cirrhosis. Although only 3.9% of children and 5.4% of adults with CF suffer from liver disease, it can be a fatal condition, with 4% of the CF population succumbing to liver disease in 2013 [12]. CF also impairs fertility, with 97% of males affected by sterility and a reduced likelihood of successful pregnancy in females [13, 14].

Animal models of CF are important as they have served to further our understanding of the mechanisms associated with CF disease progression and disease pathology and also assist in the development of new therapies to treat patients with CF. Until recently, many studies have centred on the mouse model. However, its use in understanding CF pathology is becoming less popular for a number of reasons. Firstly, mice have a short life span; therefore the progression of CF lung disease cannot be adequately studied in these animals. Of major importance, CF mice fail to develop spontaneous lung disease or chronic bacterial infections [15], unlike patients with CF. CF mice also express a CFTR-independent alternative Cl^−^ channel meaning that CFTR-deficient mice still secrete Cl^−^, thereby compensating for dysfunctional CFTR.

In light of this, researchers have moved towards animals that have similar pathological outcomes to humans in terms of CF. A CFTR knockout pig model and a pig harbouring the* ΔF508* mutation were developed in 2008 [16]. This animal is favoured for many reasons; firstly, pigs have a long life span, therefore allowing researchers to study CF disease progression over time and the efficacy of long-term therapeutics. Interestingly, even though newborn CFTR-deficient pigs have similar numbers of airway neutrophils and IL-8 levels compared to wild type (WT) pigs, they still develop spontaneous lung disease. The lungs of newborn CF piglets infected with* Staphylococcus aureus* (*S. aureus*) failed to eradicate infection, stimulating the debate as to whether airway inflammation or infection occurs first [17].

The ferret is another well-characterised animal model that is now being routinely used for CF research. CFTR expression in ferrets is identical to humans and, like pigs, they have a long life span. CFTR-deficient neonatal ferrets exhibit similar phenotypic characteristics to humans, including liver disease, pancreatic disease, and spontaneous lung infections [18]. CFTR-deficient ferrets also have lower BMI and demonstrate impaired mucus clearance [19]. Furthermore, CF ferrets fail to successfully eradicate lung infections [20] although bronchoalveolar lavage fluid (BALF) of newborn ferrets have increased levels of IL-8 and tumor necrosis factor- (TNF-) *α*, as described for humans with CF [20].

In this review we will discuss how the CF mouse, pig, and ferret models have enhanced our knowledge of CF pathology and have furthered our understanding of CF disease. This review of the literature was carried out using the MEDLINE database, Google Scholar, and The Cochrane Library databases using several appropriate generic terms.

## 2. The Effect of CFTR Mutations on Membrane Channel Abundance and Activity

**Figure 1:**
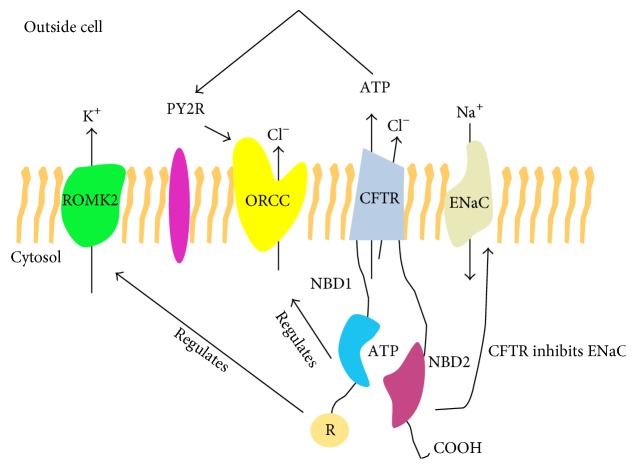
The role of CFTR in regulating additional ion channels. CFTR regulates many ion channels. CFTR primarily functions as a Cl^−^ channel. However, it also has a role in regulating the transport of K^+^ through renal outer medullar potassium channel (ROMK2). ROMK2 interacts with the intracellular cytoplasmic nucleotide-binding domains 1 (NBD1) and the regulatory (R) domain. CFTR can regulate the activity of outwardly rectified Cl^−^ channel (ORCC) through the binding of ATP to the purinergic receptor (PY2R). CFTR can also inhibit ENaC, therefore regulating Na^+^ transport into the cell.

An understanding of CFTR function, and CFTR mutations impacting on channel activity, is required prior to the description of animal models of CF-related disease. Under normal conditions the CFTR protein functions as a Cl^−^ channel pumping Cl^−^ ions out of the cell. CFTR also functions as a regulator of other ions, including Na^+^, by negatively regulating the epithelial Na^+^ channel (ENaC) [21, 22]. ENaC, also known as sodium channel nonneuronal 1 (Scnn1), is an amiloride-sensitive ion channel responsible for the transepithelial transport of Na^+^ ions. Structurally, the ENaC protein comprises three distinct *α*, *β*, and *γ* subunits encoded by the* Scnn1a*,* Scnn1b*, and* Scnn1c* genes, respectively [23]. Each subunit contains two transmembrane domains, an extracellular loop, and short N and C termini. A fourth (*δ*) subunit of ENaC has been described but remains comparably underexplored [24, 25]. Dysfunctional CFTR combined with increased ENaC activity in CF results in the inhibition of normal Cl^−^ secretion to the apical surface of airway epithelium and significant Na^+^ hyperabsorption, followed by an osmotically driven efflux of water to the basolateral membrane. The resultant dehydrated ASL volume significantly impairs mucociliary clearance [26]. In line with this, CF neutrophils have been shown to contain increased concentrations of both Na^+^ and Cl^−^ ions, secondary to impaired CFTR function [27]. ENaC impairment has also been linked to diseases beyond CF, including Liddle disease and pseudohyperaldosteronism [28]. CFTR also controls potassium (K^+^) transport by regulating the renal outer medullar potassium channel (ROMK) (Figure 1) [29].

**Figure 2:**
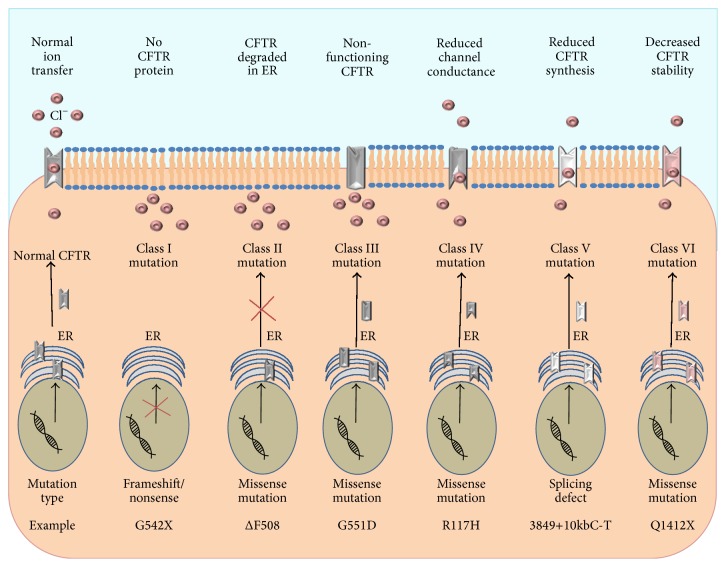
Classification of CFTR mutations. In healthy CFTR sufficient cells, the functional CFTR protein is correctly trafficked to the plasma membrane. Class I mutations result in a lack of CFTR protein synthesis. Class II mutations block CFTR processing, where misfolded protein is degraded in the ER. Class III mutations affect the regulation of the CFTR, where the CFTR channel is less functional. Class IV mutations alter the CFTR conductance of Cl^−^. Class V mutations lead to reduced synthesis of functional CFTR. Class VI mutations result in accelerated turnover of CFTR protein on the cell surface.

Over 2000 CFTR mutations have been identified to date [30] and are divided into 6 categories based on their loss-of-function effect on protein translation, cellular processing, or channel gating of CFTR (Figure 2). Missense (single amino acid change) mutations account for 39.71% of all CFTR mutations, frameshift mutations (insertions or deletions) account for 15.66%, splicing mutations (affecting the splicing of introns) account for 11.45%, and nonsense (insertion of a premature stop codon) mutations account for 8.28% of CFTR mutations worldwide [30]. Class I mutations impair protein synthesis. Premature stop codons encountered by ribosomes give rise to the early termination of CFTR protein translation. The resultant truncated CFTR, a result of a frameshift or nonsense mutation, is degraded within the endoplasmic reticulum (ER) and does not reach the cell surface [12, 31]. Class II mutations affect the maturation of the CFTR protein and are characterised by missense mutations. Improper processing of the CFTR protein causes protein misfolding. Misfolded CFTR is targeted for degradation within the ER, and thus the unstable protein does not reach the apical cell membrane. The* ΔF508* mutation is Class II mutation and is caused by deletion of a phenylalanine residue at position 508 within the CFTR gene [31]. The* ΔF508* mutation is a severe mutation and is the most common mutation with over 90% of people with CF heterozygous for the* ΔF508* mutation [12]. Class III mutations are known as gating defects and affect the opening of the CFTR channel by controlling adenosine triphosphate- (ATP-) binding and hydrolysis [31]. Although CFTR is processed correctly and is stably expressed on the cell membrane, the gating performance of this mutant is heavily impaired. The* G551D* mutation is a Class III mutation and is caused by a missense mutation at nucleotide 1784, resulting in a change from a glycine to an aspartate residue. Due to this mutation, the channel is held in the closed conformation, thus blocking Cl^−^ transport.

Classes IV, V, and VI mutations are relatively less severe than the first three classes because they still retain residual CFTR function at the cell surface. As a result of these mutations, Cl^−^ conductance may be reduced (Class IV), CFTR splicing may reduce protein function at the cell surface (Class V), or accelerated protein turnover may reduce CFTR half-life at the cell surface [12, 31, 32] (Figure 2).

## 3. CF Treatment Options

The treatment of CF involves therapeutics for management of the symptoms and potential correction of the CFTR protein. For symptomatic management of the disease, current mainstay treatments include hypertonic saline (HTS) which can be used to improve mucociliary airway clearance in patients with CF [33]. HTS consisting of 3–7% sodium chloride (NaCl) rehydrates the airways, reduces the mucosal load [34], and increases the clearance of the lung. HTS dilutes the mucus, by causing the airway cells to release water, thus restoring the moisture layer, facilitating easier removal of mucus by coughing, and reducing levels of proinflammatory mediators [35, 36]. Other treatments are also available to alleviate mucus build-up. The aerosolised mucolytic enzyme recombinant DNase (rhDNase) or its trade name Pulmozyme® cleaves the extracellular DNA that is released from cells in biofilms during necrosis, therefore reducing the viscosity of the mucus. Reducing the viscoelasticity of airway mucus allows better access of antibiotics to targeted microbes buried deep within the mucus. It is currently the third most common long-term treatment for CF patients with 46% of children and 50% of adults with CF prescribed rhDNase in 2013 [12] and has been shown to improve lung function and reduce exacerbations within 6 months [3, 37]. Maintaining good nutritional status is important for patients with CF to maximise clinical outcomes. Supplementary pancreatic enzymes, vitamins, and minerals all form part of the symptomatic control of the disease [12]. If patient health continues to decline despite all available therapeutic avenues being explored, transplantation may be considered as the next intervention. It has been shown that lung transplants increase survival compared to predicted survival for those without transplant [38].

For many years, therapies for CF have involved symptomatic control of the secondary effects of CFTR dysfunction rather than curative treatment of the primary defect. Since the discovery of the CFTR gene in 1989, considerable focus has been directed towards the development of drugs such as correctors and potentiators to address the genetic defect in CF. To this end, high throughput screening has facilitated the development of therapeutics that treat the cause of the genetic disorder rather than the symptoms. Ion channel correctors and potentiators facilitate the correction and function of the defective CFTR protein. Ivacaftor (VX-770), or its registered name Kalydeco®, is an example of a potentiator drug produced by Vertex Pharmaceuticals (MA, USA) and is currently administered orally in patients with the* G551D* mutation. Ivacaftor addressed the issues associated with the gating defects of the mutated CFTR protein. In patients with the* G551D* mutation, the CFTR is present on the cell surface but is nonfunctional; therefore ivacaftor has been designed to increase the time for which the CFTR channel remains open, allowing for the transfer of ions into and out of the cell [39]. The CFTR potentiator increased FEV_1_ by 10%, improved weight gain, reduced sweat Cl^−^, and reduced exacerbations [40]. Reports are now emerging of the potential indirect benefits of ivacaftor as an anti-inflammatory. Rowe et al. reported that CF patients treated with ivacaftor had a marked increase in mucosal bacterial clearance [41]. In support of this, Reznikov et al. demonstrated a dose-dependent reduction of* S. aureus* with ivacaftor therapy* in vitro* [42]. Furthermore, Pohl et al. highlighted the importance of ivacaftor treatment for improved neutrophil degranulation [27]. Finally, Bratcher et al. identified the potential of ivacaftor to reduce CD11b expression on isolated neutrophils from patients on treatment [43].

The* ΔF508* mutation is the most common and severe CF mutation worldwide [30]. Ivacaftor administered to patients harbouring the* ΔF508* mutation did not induce improvements in FEV_1_ or sweat Cl^−^ when compared to patients receiving placebo. Unlike the results observed for the* G551D* mutation, the potentiator did not directly repair the specific underlying defect of Class II mutations [44]. In contrast, corrector-type therapies enable the trafficking of the CFTR to the plasma membrane and have the potential to treat patients with the* ΔF508* mutation. Lumacaftor (VX-809) is an approved investigational corrector aimed at targeting the defective* ΔF508 *mutation. A combination approach of the corrector VX-809 and potentiator VX-770 drug in phase 3 clinical trials demonstrated marginally improved lung function, reduced pulmonary exacerbations, and increased BMI in patients homozygous for the* ΔF508* mutation [45, 46]. This is a promising result and could improve the quality of life for the majority of patients with CF worldwide. On July 2, 2015, the Food and Drug Administration approved the combined lumacaftor/ivacaftor approach, known as Orkambi®, to treat* ΔF508* homozygote patients. This was a major breakthrough for CF patients, and although studies have demonstrated marginal effects, the minimal positive clinical outcomes will no doubt improve the quality of life of patients [47].

## 4. The Use of Murine Models in the Study of CF

The characterisation of the CFTR gene in 1989 by positional cloning [2, 48] heralded new insights into the pathophysiology of CF disease and, merely three years after its discovery, the very first murine models of CF disease were described [49]. With an overall amino acid sequence homology of 78% between murine and human CFTR, these models have facilitated significant strides in our understanding of this complex and life-limiting disease [50]. The loss-of-function null murine model was first proposed as a model for CF by Snouwaert et al. at the University of North Carolina, in which they successfully generated a CFTR knockout (CFTR^tm1UNC^) mouse from embryonic stem cells by insertion of a stop codon in exon 10 of the CFTR coding sequence, termed the* S489X* mutation [49]. These models were further developed upon by others to generate many knockout models on various genetic backgrounds including the CFTR^tm1CAM^, CFTR^tm1HSC^, CFTR^tm1BAY^, and CFTR^tm3BAY^ models [51–54]. In addition to the knockout model of CF, various knock-in mutants were also developed. Specific CF-causing mutations were introduced to the endogenous mouse CFTR gene to bear mice carrying the Class II* ΔF508* (CFTR^tm1EUR^, CFTR^tm1KTH^, and CFTR^tm2CAM^) and* G480C* (CFTR^tm2HGU^) mutations [55–58], followed by mutants carrying the Class III* G551D* mutation (CFTR^tm1G551D^) [59]. However, intramutant variations in the survival, disease severity, and pathology of these animals have been reported. A list of CF mouse models is outlined in Table 1.

Although not a typical CFTR murine model, the “*β*-ENaC” mouse model has also been considered. As previously mentioned, the channel, in concert with CFTR-mediated Cl^−^ secretion, plays an important role in the proper regulation of ASL volume and in the adequate clearance of inhaled microorganisms and environmental particulates by the mucociliary escalator [65, 66].

### 4.1. Respiratory Tract Disease-Related Changes

As previously mentioned, CF airway disease in humans is characterised by dehydrated mucus, inflammation, and infection; and, accordingly, any mouse models used to study the disease should be representative of this [9, 67]. Where data is available, the nasal epithelium of most mouse models, including CFTR^tm1EUR^, CFTR^tm2HGU^, CFTR^tm1HGU^, CFTR^tm1HSC^, CFTR^tm1KTH^, and CFTR^tm1G551D^, mirrors the abnormal nasal electrophysiological profile seen in humans with CF with significant Na^+^ hyperabsorption and increased amiloride-sensitive basal nasal potential difference (PD) compared to non-CF littermate mice [54–56, 58–60].

While the upper respiratory tract of the murine models is representative of the upper airways of humans with CF, the lower airways represent an entirely different picture. No CFTR mouse model developed spontaneous lung inflammation without challenge, limiting their usefulness in the study of pulmonary disease progression in CF. No lower airway epithelial abnormalities were reported for knockout models including CFTR^tm1UNC^, CFTR^tm1CAM^, CFTR^tm1BAY^, and CFTR^tm1HGU^ or in models carrying* ΔF508* and* G551D* alleles such as CFTR^tm1KTH^, CFTR^tm1EUR^, or CFTR^tm1G551D^ [49, 51, 52, 55, 56, 59, 60]. The lack of severe spontaneous lung pathology in these mouse models has been partially attributed to the expression of a non-CFTR calcium-activated Cl^−^ channel (CACC) in certain mouse tissues and the fact that this expression serves to rectify the ion imbalance underlying CF lung disease. Of note, a congenic strain of the CFTR^tm1UNC^ mouse model, termed B6-CFTR^tm1UNC^, in which the murine-expressed alternative Cl^−^ channel was absent, did develop spontaneous lung disease including impaired mucociliary clearance and tissue fibrosis even when bred in a pathogen-free environment. These congenic mice also displayed impaired control of* Pseudomonas aeruginosa* (*P. aeruginosa*) infection [62, 68, 69]. The development of chronic pulmonary inflammation and bacterial persistence has been reported in the CFTR^tm1UNC^ model following intranasal challenge with* Burkholderia cepacia* (*B. cepacia*; BC7), with increased neutrophil counts and cytokine levels in BC7-challenged mice compared to control animals [70]. Using a different approach, significant pulmonary inflammation and associated pathology were also induced in S489X mice following transtracheal delivery of* P. aeruginosa*-embodied agarose beads [71]. Furthermore, low ATP12A proton-pump expression levels in CF mice may allow for normal ASL pH and unimpaired airway host defences. Such findings may explain why CF mice exhibit increased protection from pulmonary infection [72]. Interestingly, in the *β*-ENaC mouse model, excessive ENaC activity, specifically an overexpression of the *β*-subunit of ENaC (*β*-ENaC), accelerated Na^+^ absorption and induced a CF-like pulmonary clinical phenotype, including ASL depletion, significant airway mucus obstruction, and neutrophil-dominated inflammation, rendering it an important and relevant model for the study of CF lung disease. The *β*-ENaC mouse model not only demonstrated the critical importance of the *β*-subunit for optimal ENaC activity but also highlighted the mechanism between abnormal ion transport and ASL height and impaired mucociliary clearance in CF [23, 73].

### 4.2. Development of Intestinal Disease in CF Murine Models

As mentioned, CF encompasses many extrapulmonary manifestations in humans, including GI tract complications. Studies have documented significant CFTR expression throughout the GI tract, suggesting an important role for the ion channel in this milieu. Indeed, dysfunctional CFTR in the GI tract can lead to retained mucus secretions, MI, distal intestinal obstruction syndrome, intestinal dysmotility and dysbiosis, pancreatic insufficiency, and gastric reflux [11, 49, 74]. MI can develop* in utero* but is rarely life-threatening with vigilant screening and prompt surgical intervention available. Similarly, severe intestinal obstruction has been reported in many CF mouse models but unlike, in humans, develops postnatally and is associated with significant mortality. In fact, the intestinal complications exhibited by the CF murine models represent the most pronounced pathology of this model, rendering it an appropriate model in which to study CF intestinal disease [75, 76].

While variation in the degree of severity of intestinal disease exists, most models mirror the pathology reported for CFTR^tm1UNC^ and CFTR^tm1BAY^, with severe intestinal complications including runting, goblet cell hyperplasia, mucus impaction, obstruction, crypt dilation, and death a common theme [49, 52–54, 63]. Interestingly, the CFTR^tm1EUR^ and CFTR^tm2HGU^ models (carrying the Class II* ΔF508* and* G480C* mutations, resp.) display intestinal abnormalities; however the mice do not suffer the same lethal intestinal obstruction observed in humans or in the knockout mice [55, 58]. Similarly, the CFTR^tm1HGU^ mouse model displays a considerably mild intestinal phenotype which is likely explained by the “leaky” nature of the exon 10 insertional mutation resulting in residual CFTR function [77]. A second* ΔF508* model, CFTR^tm2CAM^, develops significant mucus impaction in the small intestine, jejunum, and colon with a mortality rate similar to that of the knockout models [57]. While the* G551D* model did portray some intestinal abnormalities, including obstruction, abnormal or absent villi, and necrotic and faecal material deposition in the small intestine, mortality rates were significantly lower than in the knockout murine model [59]. The survival and phenotypic differences observed between models likely result from variations in genetic background, pathogen exposure, and husbandry conditions used during the respective studies [50].

### 4.3. Pancreatic Disease in the CF Mouse Model

Another important facet of CF disease is severe structural and functional abnormalities of the pancreas, where CFTR plays a role in pancreatic duct secretion. However, murine models fail to develop pancreatic dysfunction with the same severity that humans do [11, 67]. The null knockout models such as CFTR^tm1UNC^ and CFTR^tm1CAM^ were reported to have only mild to moderate pathological changes to the pancreas, including slightly enlarged acini, luminal dilatation, or blockage of some pancreatic ducts compared to WT [49, 51]. Of note,* ΔF508* and* G551D* mouse models (CFTR^tm2CAM^, CFTR^tm1EUR^, CFTR^tm1KTH^, and CFTR^tm1G551D^) lack any pancreatic abnormalities at all [55–57, 59]. The significant disparity between the severe CF pancreatic phenotype observed in humans and the milder presentation in mice may be explained by very low levels of residual CFTR activity, sufficient to maintain ion secretion, in the pancreas of mice. Furthermore, the action of the alternative CFTR-independent CACC in the murine pancreas may result in more normalised Cl^−^ conductance and an improved pancreatic pathology [63, 67, 68].

### 4.4. Hepatobiliary Disease Development in Murine Models of CF

Liver disease has not been studied extensively in mouse models of CF disease. While liver abnormalities such as biliary cirrhosis were seen in some CFTR^tm1G551D^ mice [59], other models including CFTR^tm1UNC^ and CFTR^tm1EUR^ report no remarkable liver abnormalities [49, 55]. In humans, however, the onset of liver disease presents later in life, so, despite most mouse models displaying little or no obvious liver pathology, studies in older mice are warranted to elucidate the potential progression of this disease [62, 64]. Interestingly, two mouse models, CFTR^tm1UNC^ and CFTR^tm1G551D^, were found to have distended/ruptured and bile-laden gallbladders with concurrent neutrophil-dominated localised inflammation, suggesting a potential role for these models in investigations into the formation and potential treatment of gallstones, which present frequently in humans with CF [49, 59, 62]. This is in contrast to the CFTR^tm1KTH^ in which no obvious gallbladder pathology was described [56].

### 4.5. Fertility and Reproductive Problems in the CF Mouse

Infertility is a common characteristic of CF affecting approximately 97% of males due to a congenital absence of the vas deferens resulting in azoospermia [14, 78]. However, many mouse models, including CFTR^tm1CAM^ and CFTR^tm2HGU^, fail to recapitulate this infertility [49, 51, 52, 55, 58, 59]. The lack of observed corollary between mouse models and humans is speculated to again involve the expression of the aforementioned alternative Cl^−^ channel in murine seminal vesicles and epididymides [79]. Although some females with CF are also affected by infertility, studies are far less numerous. Mouse models have been useful in the past to study reproductive patterns in females. A knockout model, CFTR^tm1UNC^, and the* ΔF508* mouse model, CFTR^tm1KTH^, have been used to demonstrate impaired sperm transport within the CF female mouse reproductive tract, due to inadequate fluid control. This study also reported delayed puberty, smaller reproductive organs, and decreased oocyte numbers during ovulation, which may all contribute to female infertility in humans [67, 80].

### 4.6. The Use of the CF Murine Model for Evaluating New Treatment Options

The development of murine models birthed a new avenue in which to test experimental compounds for their efficacy in targeting the dysfunctional CFTR gene. Du et al. generated a mouse model carrying the Class I mutation* G542X* in which they demonstrated the ability of the orally bioavailable PTC124 compound to read through premature stop codons, a key feature of Class I CFTR mutations, resulting in partial restoration of the CFTR protein in the mouse intestinal tract [81]. More recently, a similar read-through compound, NB124, was shown to suppress nonsense mutations and partly restore CFTR function in a* G542X* transgenic mouse model [82]. Other drugs and gene therapy approaches have also been described in murine models [83–85].

Aside from the CFTR modulator approach, ENaC channel antagonists are also being investigated using the *β*-ENaC mouse model for their efficacy in restoring normal Na^+^ flux and their downstream effects on mucus clearance and inflammation [86].

## 5. The Use of Pig Models to Study CF Pathology

Although the field of CF research has been much advanced through the use of murine models over the past two decades, the manifestations seen in mice do not fully mirror the normal clinical phenotype of CF in humans, particularly in relation to pulmonary disease progression. Consequently, researchers have turned their attention to the porcine model of CF disease as a more robust representation of the human CF phenotype [75]. Indeed, the pig shares 92% CFTR sequence homology with human CFTR [87]. In 2008, Rogers and colleagues generated a pig model of CF in domestic animals (*Sus scrofa*) using recombinant adenoassociated virus-mediated (AAV-mediated) gene targeting in foetal pig fibroblast. This process involved the generation of two different CFTR defects including the null allele, which lacked production of CFTR protein, and the heterozygous* ΔF508* mutation [16]. Furthermore, in 2011, Ostedgaard and coworkers employed the heterozygous CFTR^ΔF508/ΔF508^ pig previously generated by Rogers et al. (2008) [16] to generate homozygous CFTR^ΔF508/ΔF508^ animals. Maintenance of these animals involves initial surgery to alleviate intestinal obstruction [88]. CF piglets also require pancreatic enzyme replacement therapy, vitamins and H2 blockers, and/or proton-pump inhibitors to improve gastric acid control [87, 89].

### 5.1. Respiratory Tract Disease-Related Changes in the Porcine CF Model

The pig model was a suitable candidate to study CF lung disease due to anatomical and physiological similarities between the human and porcine lung [90, 91]. Interestingly, as far back as 1976 the respiratory tract of pigs was used to mimic the cycle of infection and inflammation of humans [92]. Phenotypically, Meyerholz et al. demonstrated that the trachea and bronchi of CF piglets were smaller than WT piglets [93], although the lung volume was reportedly similar to their non-CF littermates. CF piglets (CFTR^−/−^ and CFTR^−/ΔF508^) have also been shown to have increased airway resistance, indicating airway obstruction [94] and airway wall thickening, similarly to children with CF [17]. Additionally, Ostedgaard et al. reported that CFTR^ΔF508/ΔF508^ piglets had airways disease from as young as 2 days old, with the development of mucopurulent material causing airway obstruction [87].

In recent years in the CF field, a debate has developed as to whether inflammation or infection occurs first in the CF airways, with some authors agreeing that inflammation precedes infection in children with CF [95, 96] and others providing evidence to the contrary [97]. The pig model of CF provided scientists with a strong platform to study this important conundrum in detail. Studies employing the CFTR^−/−^ pig demonstrated similar numbers of immune cells and cytokine IL-8 concentrations in WT and CF piglets [89]. Building on this foundation of knowledge and because CFTR^−/−^ pigs lack inflammation at birth, Chen et al. concluded that this was an ideal model to study electrolyte transport defects. Results demonstrated that c-AMP-stimulated Cl^−^ transport was impaired in nasal and tracheal transepithelial airways of CF piglets with the CFTR^−/−^ mutation [98], similar to humans with CF [99, 100]. Moreover, hyperabsorption of Na^+^ in nasal cells of CF piglets was increased when compared to WT piglets.

Of major importance, BALF from CFTR^−/−^ piglets contained greater numbers of bacteria, including* S. aureus*, which was not present in the WT piglets [17]. Interestingly, this data reflects reports in children with CF, where the main bacterium isolated from airway samples was* S. aureus* [4]. Moreover, bacteria were isolated from the lungs of CFTR^ΔF508/ΔF508^ piglets, in contrast to WT piglets, which lacked culturable bacterial colonies [87]. This data provided the first line of evidence that inflammation occurs after infection in CF piglets and demonstrates that an inherited defect associated with a lack of CFTR protein and function results in impaired bacterial clearance and development of inflammation. Collectively, these reports indicate that CFTR^−/−^ and CFTR^ΔF508/ΔF508^ pig models resemble similar lung pathological outcomes as their genotypically identical human counterparts and facilitate our understanding of lung disease in CF, providing evidence on the origins of inflammation and infection within the lungs.

### 5.2. Intestinal Disease Development in the CF Pig Model

MI is classified as either simple or complex. Simple MI is the failure to remove the meconium within the first 48 hours of birth and complex MI represents intestinal atresia, necrosis, and perforation [11]. Interestingly, using the CFTR^−/−^ pig, Rogers et al. demonstrated that the earliest manifestation of CF was MI [89]. Studies have shown that 100% of CFTR^−/−^ and CFTR^ΔF508/ΔF508^ pigs develop complex MI [87, 89, 101]. This is in agreement with the increased occurrence of MI in humans carrying the* ΔF508* mutation [102].

No inflammation was noted in the intestines of CFTR^−/−^ piglets; however the spiral colon of the CFTR^−/−^ pig contained microcellular accumulations and high levels of mucus [87]. CFTR^−/−^ piglets also had intestinal atresia, a complication that occurs at birth resulting in the narrowing of the intestine. Interestingly this also commonly occurs in CF infants [101]. Ostedgaard et al. reported that CFTR^ΔF508/ΔF508^ pigs had similar GI tract pathology to the CFTR^−/−^ pigs [87]. Additionally, diverticulitis a condition caused by the formation of a sac within the colon wall commonly occurs in CF individuals and was also reported in CFTR^−/−^ piglets [103]. These findings indicate that the GI pathologies of CFTR^−/−^ and CFTR^ΔF508/ΔF508^ are similar, indicating a CFTR-related defect affecting pathological outcomes in piglets, not dissimilar to their human counterparts.

### 5.3. Pancreatic Disease in the CF Pig Model

Data has shown that CFTR^−/−^ piglets fail to gain weight [89]. Pancreatic insufficiency is another characteristic associated with CF piglets and is phenotypically similar to CF infants [104]. Studying pancreatic disorder in adult CF individuals is difficult, as it involves sampling the fluid of the jejunum which contains a mixture of bile and pancreatic fluid, as the common bile duct and pancreas duct intervene. Interestingly, pigs have a separate common bile and pancreas duct, allowing researchers to study bile and pancreatic fluid separately [105]. CFTR^ΔF508/ΔF508^ pigs presented with decreased pancreatic fluid secretions of altered pH and protein content [105]. In CFTR^−/−^ pigs, the number of acinar cells and cytoplasmic zymogen granules decreased, with increased zymogen secretions found in the interstitium and within pancreatic ducts; this is similar to what is described in adults with CF [101]. Of interest, histology of the pancreas of CFTR^ΔF508/ΔF508^ pigs was less severe than the CFTR^−/−^ pigs [87] indicating that minimal CFTR presence/function in* ΔF508* pigs may reduce pancreatic disease progression, an important finding which may facilitate researchers in understanding pancreatic disease development and progression in CF individuals.

### 5.4. Hepatobiliary Disease in the Pig Model of CF

Within the hepatobiliary system, the CFTR is located on the apical membrane of cholangiocytes [106] and plays a role in regulating fluid and electrolyte stability in bile. Gallbladder abnormalities, including a noticeably smaller gallbladder, occur in approximately 10% to 30% of all patients with CF [107]. Indeed, congealed bile and mucus have been found in smaller gallbladders of CFTR^−/−^ and CFTR^ΔF508/ΔF508^ pigs compared to WT pigs [87, 89]. It was shown that the CFTR^−/−^ pig had mild to moderate hepatic lesions [89] and elevated bile protein compared to the WT pigs [105]. Moreover, the CFTR^−/−^ pig had increased cellular inflammation, ductal hyperplasia, and mild fibrosis. In concurrence, the CFTR^ΔF508/ΔF508^ pig model presented with inflammation and exhibited common symptoms of biliary cirrhosis [87].

### 5.5. The Potential Use of the CF Pig Model for Development of Novel Therapeutics

Cook et al. demonstrated, using pig epithelial cells, that ivacaftor therapy functions to potentiate the CFTR channel, similar to human epithelial cells [108]. This provides researchers with the opportunity to study the longevity of ivacaftor therapy on pig models and further opens the research to other potentiators and correctors.

## 6. Disease Phenotype of the CF Ferret

The ferret model has been extensively used in the assessment of lung infections including severe acute respiratory syndrome [109] and influenza virus [110] due to the similarity in lung cell biology and anatomy to humans, thus making it an ideal candidate for CF research. The extent of lung similarities between humans and ferrets includes comparable submucosal gland expression of CFTR within the serous tubules, important for the secretion of fluids and mucus into the lung [111] and for protection of the airways from infection [112]. Moreover, CFTR pharmacologic and bioelectric functionality in ferret epithelia is similar to that of human epithelia [113]. The ferret's short gestation period and time to reach adolescence within 4–6 months along with its smaller size compared to the pig may make it a more cost-effective model to maintain. The development of the CF ferret model has utilised recombinant adenovirus gene targeting of the CFTR gene to introduce partial disruption of exon 10 or complete deletion of the CFTR gene [114]. This process has generated CFTR^+/−^ breeding pairs for the production of CFTR-deficient ferrets [18].

### 6.1. Respiratory Tract Disease-Specific Changes in the Ferret Model of CF

Of the CFTR^−/−^ ferret kits that avoid MI-induced death, lung infections in the form of pneumonia and poor nutritional status are the main causes of mortality. Even with improved weight gain, CFTR^−/−^ ferrets demonstrate increased levels of enteric bacteria in their lungs, with* Staphylococcus* spp. and* Streptococcus* spp. isolates found. CFTR-deficient ferrets demonstrated improved survival with the administration of antibiotics. Consistent with CF individuals, the CFTR^−/−^ ferret presents with dysfunction of the proximal airways including impaired submucosal gland fluid secretion and c-AMP-induced Cl^−^ permeability [18].

### 6.2. Intestinal Disease Studied in the CF Ferret Model

In CF ferrets, intestinal obstruction in the form of MI is found in approximately 75% of CFTR^−/−^ ferret kits [18], compared to 13%–17% observed in human CF neonates [10]. CFTR^−/−^ ferrets show greater morbidity after 48 h as they fail to thrive with half of the MI-positive ferret kits succumbing to intestinal lesions of the colon or ileum [18]. Of approximately 25% of newborn kits that survive MI, none thrive or live longer than 4 days. The ferret intestinal tract demonstrates a more severe form of MI compared to humans, mice, and pigs [18] due to the ferret's faster intestinal transit time which results from the lack of a cecum seen in omnivores or herbivores [115].

### 6.3. Pancreatic and Hepatobiliary Disease and Fertility

The pancreas of the CFTR^−/−^ ferret demonstrates a similar histopathology to that of human neonates where lesions and dilation of acini and ductules occur [18, 116]. CF ferrets that survived MI demonstrated increased pancreatic inflammation with exocrine tissue loss similar to that observed in humans with CF. Sun and colleagues administered a proton-pump inhibitor, omeprazole, to CFTR^−/−^ ferrets to counter the lower-than-normal gut pH as a result of impaired pancreatic secretions of bicarbonate. This improved fat absorption and resulted in weight gain comparable to the CFTR^+/+^ ferrets [18].

The gallbladder and liver of CFTR^−/−^ ferret kits demonstrated no histological differences between the CFTR^+/+^ or CFTR^+/−^ kits. Through blood analyses the liver function of CFTR^−/−^ ferret kits demonstrated low levels of cholesterol and high levels of plasma alanine aminotransferase (ALT) and bilirubin, which highlights an early onset of liver disease, although no liver lesions were found in CFTR^−/−^ ferrets. CF infants that present with liver disease demonstrate a similar pathology to that observed in ferret neonates although it commonly resolves within humans [117].

CFTR^−/−^ ferrets also demonstrate reduced bile acid absorption by the GI tract, causing poor fat absorption leading to reduced nutritional status [18]. The CFTR^−/−^ ferrets poor nutritional condition results from poor absorption of nutrients which is corrected by supplementation of ursodeoxycholic acid (UDCA) which normalises the levels of liver ALT and bilirubin. However, even in combination with antibiotics to stop infections of the lung, mortality still occurs within 5–9 days [18].

Absent or degenerate vas deferens was found in CFTR^−/−^ ferrets similar to that of humans [118]. In CF humans, the vas deferens is degenerated due to mucoid blockage and a progressive degeneration [119]. Ferrets kits with either CFTR^+/+^ or CFTR^+/−^ demonstrated intact normal vas deferens in the same study.

## 7. Concluding Remarks

The generation of CF animal models has greatly advanced our knowledge of the disease and will continue to inform upon the complexity of this disease for many years to come. Inherent weaknesses lie in some earlier models but the generation of larger models endeavoured to address these deficiencies. The phenotypic characterisations of the three models discussed herein are outlined in Table 2.

There are many advantages to the use of mice to study the pathology of CF disease, including the ease with which they can be genetically manipulated, short gestation times, and low cost of animal upkeep [62]. However, there are also a number of limitations to their use. Firstly, phenotypic variations exist between models and, indeed, the degree of severity of such manifestations also differs greatly between models. While the mouse model certainly displays severe intestinal obstruction and altered nasal electrophysiology similar to that seen in humans, the mouse models fail to initiate spontaneous lung infection without pathogen challenge. Most models only display mild complications in the pancreas, liver, and vas deferens, which is in contrast to the severe abnormalities in human tissues. It is suggested that low levels of residual CFTR expression may induce sufficient ion transport to alleviate the severity of the disease in the mouse model. Also, in mice, the epithelial expression of a second Cl^−^ ion channel with an alternative signalling pathway to CFTR may compensate for dysfunctional CFTR and may account for the phenotypic disparities between the human and murine manifestations of CF disease [64, 68].

Pigs are ideal models for the study of CF for a number of reasons; they have perfect reproductive characteristics with a large number of offspring. Furthermore, they have a fast maturation rate and have a long life span, thus allowing researchers to study the pathological outcomes and prognosis of CF. A lack of CFTR protein and function in CFTR^−/−^ piglets impacts on the lungs, liver, pancreas, and GI tract, all organs which are similarly affected in humans with CF [87, 89, 98]. However, MI presents in 100% of CFTR^−/−^ piglets (in contrast to its prevalence of 15% in infants with CF) and is fatal without early surgical intervention [89]. This burden may limit the usefulness of the porcine model in some institutions. The potential risks involved in surgery and complications associated with MI, including intestinal atresia, demand great understanding and commitment to the use of these animals in the study of CF.

The ferret model does demonstrate a large number of similarities to CF pathology in humans, particularly neonates. Ongoing steps to generate a CFTR^−/−^ ferret that overcomes the GI pathology to enable animals to reach adolescence are needed to establish whether ferret lung pathology mirrors that of humans with CF. Nevertheless, it must be mentioned that the appearance of lung infections at both the start and end of the CFTR^−/−^ ferret life span mirrors human disease, highlighting the potential benefits of this model. The development of* ΔF508* and* G551D* specific mutant is required to establish the ferret as an exemplary animal model for CF.

In summary, it is certain that animal models have significantly contributed to our understanding of this complex disease. The animal models now available will continue to be modified and improved to provide an even greater depth to our knowledge. Importantly, animal models will not only provide information on the pathology of disease but also provide an invaluable canvas on which to test new compounds and therapies for use in CF disease management.

## Figures and Tables

**Table 1:** Mouse models of cystic fibrosis.

Identifier	Mutation	Detectable CFTR mRNA	Salient features	Reference
CFTR^tm1UNC^	Exon 10 replacement	No detectable WT CFTR mRNA	Severe intestinal complications	[49]

CFTR^tm1CAM^	Exon 10 replacement	No detectable WT CFTR mRNA	Pancreatic ductal blockage Severe intestinal pathology	[51]

CFTR^tm2CAM^	*ΔF508* Exon 10 replacement	Mutant mRNA 30% of WT CFTR levels	No pancreatic abnormalities Longer survival than null models	[57]

CFTR^tm1BAY^	Exon 3 insertional duplication	<2% of WT CFTR mRNA	Severe intestinal complications	[52]

CFTR^tm3BAY^	Exon 2 replacement	No detectable WT CFTR mRNA	Severe intestinal complications	[53]

CFTR^tm1EUR^	*ΔF508* Exon 10 insertion (hit and run)	Mutant mRNA at normal WT levels	Nonlethal intestinal abnormalities; no pancreatic or liver abnormalities	[55]

CFTR^tm1KTH^	*ΔF508* Exon 10 replacement	Decreased mutant mRNA in intestine	Impaired sperm transport within the female reproductive tract; no gallbladder pathology	[56]

CFTR^tm1HGU^	Exon 10 insertion	10% of WT CFTR mRNA	Mild intestinal complications; longer survival	[60, 61]

CFTR^tm2HGU^	*G480C* Exon 10 insertion (hit and run)	Mutant mRNA at normal WT levels	Nonlethal intestinal abnormalities	[58]

CFTR^tm1HSC^	Exon 1 replacement	No detectable WT CFTR mRNA	Severe intestinal complications	[54]

CFTR^tm1*G*551*D*^	*G551D* Exon 11 replacement	Mutant mRNA 53% of WT CFTR levels	Absent or mild (nonlethal) intestinal obstruction	[59]

CFTR: cystic fibrosis transmembrane conductance regulator; WT: *wild type*.

Adapted from [62–64].

**Table 2:** Phenotypic manifestations of cystic fibrosis in humans and animal models.

	Spontaneous lung infection	Pancreatic disease	Intestinal disease	Liver and gallbladder disease	Reproduction
Human	Yes	PI	MI	Biliary cirrhosis	Severe vas deferens defect
Mouse^*∗*^	No	No	Intestinal obstruction, often fatal	No	Reduced fertility in females
Pig	Yes	PI	100% MI	Biliary cirrhosis	Severe vas deferens defect
Ferret	Yes	PI	75% MI	Liver disease	Severe vas deferens defect

^*∗*^The phenotypic manifestations outlined above may differ between mouse models. PI: *pancreatic insufficiency*; MI: *meconium ileus*.
